# Machine learning and personalized nutrition: a promising liaison?

**DOI:** 10.1038/s41430-023-01350-3

**Published:** 2023-10-13

**Authors:** Paola G. Ferrario, Kurt Gedrich

**Affiliations:** 1 Department of Physiology and Biochemistry of Nutrition, Max Rubner-Institut, Karlsruhe, Germany; 2 Technical University of Munich, ZIEL – Institute for Food & Health, Research Group Public Health Nutrition, Freising, Germany

**Keywords:** Computational models, Nutrition

## Introduction

Personalized nutrition (PN) receives growing attention by the research community, the food industry, the media, and the public. New scientific papers are published and position statements, research calls, and commercial offers appear at rapid pace.

In fact, PN started as a new scientific discipline 20 years ago when the human genome became available [1] and first commercial offers for PN also emerged in 2003. PN meanwhile matured and is nowadays more than gene-based dietary recommendations. Why is PN currently experiencing increased interest? Although there are various reasons [2], one particular is that new technologies became available that substantially ease the collection of individuals’ data. These cover measurements of biochemical, vital, and lifestyle parameters that can be collected not only more precise, but also faster, cheaper and at higher frequencies. But how can we turn those data into meaningful information? How can patterns and trends be extracted from the data flow? And, how can the data be used for PN?

Currently, there are numerous statistical approaches available to analyze and interpret such large amount of data. In particular, machine learning - an umbrella term referring to a wide number of statistical approaches - offers tools to gain knowledge from data.

## Machine learning

Machine learning (ML) is about *learning from data*, i.e. relating predictors to an outcome. Is there a functional relationship describing the dependence structure between predictors and outcome? Should such a functional relationship exist but be unknown, ML can be used to reveal complex interdependencies. Its general approach is to split the available data into two subsets. A training set, in which the observed predictors and the outcome are used to develop a so-called prediction model, or learner. In other words, algorithms are used on the training dataset to explore the assumed but unknown dependence structure, which enables the prediction of the outcome for new, further values of predictors. The final goal, however, is not to just describe the relationship between the predictors and the outcome in the training dataset, but rather, to find a good prediction rule for the outcome for further predictors values [3]. The quality of the prediction is assessed by applying the learner on the second subset of the available data, the so-called test dataset. A good learner will accurately predict the outcome based on the available predictors. Here, the term “machine” emphasizes that the outcome is automatically assigned. Just to give a few examples, the following methods count among the ML methods: linear regression, support vector machines, decision trees, and random forest [4].

## Machine learning for personalized nutrition

ML techniques have already been used in the field of PN; a systematic review of ML in PN identified 60 relevant papers published between 2014 and 2021 [5].

While PN originated from the integration of genetic information for generating personalized dietary recommendations, it now also integrates data from other “omics” domains, such as epigenomics, metabolomics, or microbiomics as well as other data, such as vital and lifestyle parameters. PN approaches somehow become data-greedy. But the more “personalized” nutritional advice is, the larger the sample size needs to be. Otherwise, the so-called “*p* > *n*” problem arises (where *p* is the number of predictors and *n* is the sample size).

In fact, having in mind linear (mixed) models as standard statistical approach to build a “learner”, such models cannot be fitted in case *p* + 1 is greater than n. Mathematically speaking, such a fit corresponds to a system of equations with more unknowns than equations, which is a non-solvable equations system [6]. Consequently, if the number of predictors exceed the sample size, i.e. *p* > *n*, some ML techniques become inapplicable. One possible solution consists in using variable-selection techniques. Other options, for instance, are so-called penalized regression models such as lasso and ridge regression [4]. When working with variable-selection techniques, it is important to perform variable selection on the training dataset and not on the whole dataset [6] to avoid overfitting and overestimating prediction performance.

Generally, PN approaches are often justified based on the fact that individuals respond to nutritional interventions in a heterogeneous manner. Often, the outcome is not a continuum, but rather shows certain subgroups of individuals reacting differently to the same interventions. In order to identify heterogeneous intervention effects, a subgroup analysis can be conducted. Specifically, statistical analyses based on an entire dataset are repeated on subsets of the data. Those subsets are typically identified using baseline information: For continuous covariates, subgroups are defined by (pre-defined) cut-offs, such as the BMI categories defined by WHO (WHO Consultation on Obesity (1999: Geneva, Switzerland) & World Health Organization. (2000). Obesity: preventing and managing the global epidemic: report of a WHO consultation. World Health Organization). The result of the subgroup analysis is the identification of subgroups of individuals reacting in a similar manner [7]. Another possible way is the estimation of individualized treatment effects, i.e., individualized risk prediction [8]. In this context, if the treatment effect varies across subpopulations, it could be of interest to consider so-called Individualized Treatment Rules (ITR). The ITR assigns treatments favoring one treatment over alternative treatments, where the choice of a proper treatment is made for achieving optimal outcomes. The optimality is quantified by the so-called population average outcome, for instance [9]. The decision for one or another treatment is made according to some individuals’ baseline characteristics. Specifically, linear mixed models are fitted with baseline covariate-treatment interaction terms. Both of these approaches can be addressed with ML techniques [10]. Specifically, random forest analyses could be useful [11] in constructing decision trees, of which the final leaves constitute subgroups of individuals with similar responses.

## Opportunities, limitations, and challenges

ML can be applied to address different questions in the field of PN and it is predicted that the coming years, studies applying ML to PN will increase substantially [5]. This will be driven by the development of new and better technologies together with additional progress in computing power. Some institutions – such as the National Institutes of Health in the US - provide strong financial support for the development of algorithms for PN (https://www.nih.gov/news-events/news-releases/nih-awards-170-million-precision-nutrition-study, 2022).

Although this area of research is vibrant and produces novel results, it is important to emphasize that most studies conducted so far are descriptive in nature [12]. They do not provide conclusions as to whether personalized dietary recommendations are more effective than generic recommendations, that needs to be scientifically proven.

Another crucial aspect concerns the reproducibility and the replicability of machine learning studies in the PN context. Common definitions of these two terms are as follow. A study is considered being reproducible if another researcher is able to duplicate the results of this study using the same raw data, the same analysis files, etc. Indeed, a study is said to be replicable if it is possible to draw similar conclusions after having performed the same experiments and analyses but for new data [13].

However, there is still no consensus on these definitions, which is particularly true throughout different research fields, such as ML and PN. Moreover, the discussion around reproducibility and replicability is typically restricted to traditional statistical methods and there is a need for extension to methods like ML, and even more for ML methods used in the context of PN. How good is the study replicability if intervention effects are subtle and down to the individual? How good are ML algorithms in an unfavorable signal-to-noise ratio? What about the generalizability of studies in PN context? In fact, in PN sometimes the data stem from a nonrepresentative subset [14] of an elite group of individuals, who have already high knowledge of nutrition and high capacity to implement dietary recommendations.

Moreover, the application of ML in the field of PN faces still another challenge: There is a lack of valid outcome measures. Some parameters such as blood glucose levels can easily and even continuously be measured, but are they indeed suitable overall health indicators in persons without diabetes [1]? Other outcome parameters such as the composition of the gut microbiome do not even have a clear definition of an optimal status [15]. And how should ML deal with a multitude of possibly competing outcome measures such as serum levels of glucose, lipoproteins, or the soluble transferrin receptor?

Finally, PN still tends to show a strong focus on biomedical outcome parameters and often ignores social, cultural, culinary, economic, or environmental aspects of diets, which have a profound impact on the acceptability of and long-term compliance with PN recommendations [2, 16]. The integration of such additional aspects into PN algorithms, however, still aggravates the *p* > *n* problem and underpins the needs for more and better data such that ML could validly contribute to a multidisciplinary approach to true PN.
